# Stress and Coping Strategies of Nurses Working with Patients Infected with and Not Infected with SARS-CoV-2 Virus

**DOI:** 10.3390/ijerph19010195

**Published:** 2021-12-24

**Authors:** Grażyna Puto, Maria Jurzec, Anna Leja-Szpak, Joanna Bonior, Marta Muszalik, Agnieszka Gniadek

**Affiliations:** 1 Institute of Nursing and Midwifery, Faculty of Health Sciences, Jagiellonian University Medical College, 31-501 Krakow, Poland; jurzec.maria@gmail.com (M.J.); agnieszka.gniadek@uj.edu.pl (A.G.); 2 Department of Medical Physiology, Faculty of Health Sciences, Jagiellonian University Medical College, 31-126 Krakow, Poland; a.leja-szpak@uj.edu.pl (A.L.-S.); joanna.bonior@uj.edu.pl (J.B.); 3 Department of Geriatrics, Nicolaus Copernicus University in Toruń, Collegium Medicum in Bydgoszcz, 85-094 Bydgoszcz, Poland; muszalik@cm.umk.pl

**Keywords:** stress, coping strategies, pandemic, COVID-19, nurses, SARS-CoV-2 virus, healthcare workers

## Abstract

Introduction: Working during the COVID-19 pandemic is a particular challenge for nurses because, while performing their daily routines, they are exposed to physical and social consequences of the SARS-CoV-2 virus, which is accompanied by intensified stress. The aim of this study was to assess the intensity of stress and coping strategies applied by nurses working with both infected and non-infected patients with SARS-CoV-2 virus during the COVID-19 pandemic. Materials and Methods: The study was conducted between January and March 2021. Due to the epidemiological situation, the questionnaire was posted on Facebook in nurses’ groups and sent out via the “Messenger” and “WhatsApp” applications. Stress intensity was assessed by means of the Perceived Stress Scale (PSS-10), whereas coping strategies were assessed using the Mini-COPE stress coping inventory. Results: Among 151 surveyed nurses, more than half (52.3%) worked with infected patients and the remaining ones (47.7%) worked with non-infected patients. The level of stress perceived by nurses working with infected patients was higher than among nurses working with patients without SARS-CoV-2 infection (22.22 ± 5.94 vs. 20.21 ± 5.68, *p* = 0.03). The nurses working with infected patients were most likely to choose coping strategies focused on the problem (2.00 ± 0.62) and emotions (2.01 ± 0.69), whereas those working with non-infected patients usually chose strategies focused only on the problem (2.11 ± 0.58). Conclusions: During the COVID-19 pandemic, nurses working with SARS-CoV-2 patients experienced more intense stress than those working with non-infected patients. Nurses working with SARS-CoV-2 patients tended to cope with stress using strategies focused on the problem and on emotions, while those working with non-infected patients were more likely to choose strategies focused only on the problem.

## 1. Introduction

The COVID-19 pandemic has been a global challenge for healthcare workers, among whom nurses, undeniably, constitute the biggest professional group. Working in pandemic conditions is a significant challenge for nurses because, while performing their daily routines, they are particularly exposed to the physical and social consequences of the SARS-CoV-2 virus, which is accompanied by intensified everyday stress [1,2]. It is difficult for nurses to find themselves in this new reality, taking into account the speed with which the disease is spreading, an insufficient time to prepare for a record number of seriously ill patients, the high mortality rate, everyday work in personal protective equipment, unpredictability of events, loss of control, sense of helplessness, and the fear accompanying everyday work routines. These are just some of the reasons why nurses’ work is extremely vulnerable to stress and its effects during the COVID-19 pandemic [2,3,4,5,6].

As far as the concept of coping with stress is concerned, three types can be identified. The first one is problem focused. It involves activities aimed at controlling the stressor in order to limit or solve the problem. It consists of searching, processing and using all information available that can help to achieve a balance. Another one, which is emotion focused, is aimed at reducing negative emotions provoked by a stressful situation, which might increase agitation and give a greater mobility to act. The third type is avoidance focused. It aims at distracting and diverting attention from the problem and the unpleasant sensations that may accompany a stressful situation [7,8,9]. Other strategies of coping with stress depend on the situations people are in. They include positive reframing or seeking social support [7,9]. When the stress is not too strong, active coping is frequently chosen, whereas when it increases, avoidance strategies start to prevail. Denial, venting and self-distraction are often accompanied by a sense of mental discomfort [9,10]. Research on identifying differences in nurses’ work in the face of such enormous stress, such as the COVID-19 pandemic, seems to be of vital importance in order to develop diverse and highly effective intervention strategies.

The aim of this study was to assess the intensity of stress and coping strategies applied by nurses working with both infected and non-infected patients with the SARS-CoV-2 virus during the COVID-19 pandemic.

## 2. Materials and Methods

### 2.1. Data Collection

This study was conducted in Poland between January and March 2021 during the SARS-CoV-2 pandemic. From 13 January, a decrease in the number of new infections was observed (the average daily number of infections reported in the previous 7 days reached 8.5 thousand) in Poland, while from 15 January to 15 February the average daily number of new infections was 5611.

A gradual increase in the number of daily infections was observed from 17 February. According to an analysis of infections, the study was started during the flattening of the disease curve and at the beginning of another (third) wave of the COVID-19 pandemic [11].

The questionnaire was prepared in Polish using the Google Forms tool. Due to the epidemiological situation at that time, the link to the survey was published on social media pages, such as “Facebook”, “Messenger” and “WhatsApp”, which were available only to nurses working in the southern regions of Poland. Participants were encouraged to forward the survey link to their colleagues who met the survey criteria. Completing online questionnaires is considered to be an established method in healthcare studies. The process of data collecting is simplified, rapid, and ensures greater data accuracy [12,13,14]. Because of the adopted method of recruiting respondents (snowball sampling), it is not possible to estimate the exact size of the population available for the survey, the percentage of responses received or the degree to which the surveyed sample is representative of the whole population.

The criteria for participating in the study included: working as a nurse with direct contact with a patient, work experience ≥ 1 year, and informed consent to participate in the study. Exclusion criteria included non-medical staff, remote work with a patient, work experience ≤ 1 year, and lack of consent to participate in the study by checking the box “I consent to participate in this survey and I am aware of the fact that I can withdraw my consent for further participation in the survey at any time without giving any reason.” Each respondent was informed about the full anonymity of the study and the possibility of resigning from it at any time without giving a reason.

### 2.2. Participants

The survey questionnaire was completed by 156 people, but due to incomplete data, only 151 respondents were included in the research.

### 2.3. Ethical Procedures

The study was conducted anonymously on a sample of volunteers in accordance with the guidelines of the Code of Ethics of a Research Employee, as well as with the Declaration of Helsinki developed by the World Medical Association [15].

### 2.4. Study Design

The study applied a survey questionnaire that included questions dealing with demographic and social characteristics (gender, age, education, marital status, financial status), workplace during the COVID-19 pandemic (with patients infected or not infected with SARS-CoV-2 virus), and work experience in the nursing profession, monthly number of working hours, and standardized tools.

*Perceived Stress Scale (PSS-10)*

The assessment of the intensity of stress related to their life situation in the last month was carried out with the application of the Perceived Stress Scale (PSS-10). The scale consists of 10 questions that are related to subjective feelings provoked by problems, personal experience, behavior and coping strategies. The score uses a 5-point scale, where: 0—never, 1—almost never, 2—sometimes, 3—fairly often, 4—very often. The global score is obtained by adding up particular scores and can range from 0 to 40 with higher scores indicating higher perceived stress. The interpretation of the results also specified sten norms adopted for PSS-10 (low stress intensity—from 1 to 4 sten scores/0–13 scores, average intensity—from 5 to 6 sten scores/14–19 scores and high intensity—from 7 to 10 sten scores/20–40 scores). The authors who adapted the scale to Polish conditions obtained a reliability of 0.86 according to Cronbach’s alpha [10]. Research results indicate that the PSS-10 scale accurately measures subjective feelings of stress related to personal problems and the applied coping strategies.

*The Mini-COPE Coping Inventory*

The assessment of coping strategies was conducted with the application of the Mini-COPE coping inventory. The tool consists of 28 statements, which are part of 14 strategies of coping with stress, such as active coping, planning, positive reframing, acceptance, sense of humor, turning to religion, seeking emotional support, seeking instrumental support, self-distraction, denial, venting, substance use, behavioral disengagement or self-blame. Problem-focused strategies include active coping and planning and seeking instrumental support, whereas emotion-focused strategies include seeking emotional support, turning to religion or denial. Respondents choose one out of four alternatives to describe their attitudes to each statement and obtain scores according to the following rules: “I hardly ever do it”—0, “I rarely do it”—1, “I often do it”—2, “I almost always do it”—3. Each strategy is assessed separately on the basis of the average score obtained from two statements that are assigned to it. The reliability of the Mini-COPE inventory is 0.86 [10]. Mini-COPE is the most commonly used tool to describe coping strategies, i.e., it can be used in order to assess the typical feelings and ways of reacting in situations of severe stress.

### 2.5. Statistical Analysis

In a statistical analysis of obtained results, the distribution of qualitative variables is described by means of absolute numbers for particular categories (N) and their percentage share in the distribution of the variable (%). Average values of variables with a normal distribution are described using the mean and standard deviation (SD), and median values were used when the distribution was non-normal. Normality of the distribution was assessed by examination of the Q-Q plot. Relationships between qualitative variables are shown in the form of contingency tables. An analysis of the statistical significance of these correlations was conducted with the application of Pearson’s chi^2^ test when the figures in at least 80% of the table cells were greater than 5, or by Fisher’s exact test for 2 × 2 tables and Fisher–Freeman–Halton test for tables of other sizes. The comparison between nurses working with patients infected with SARS-CoV-2 virus and those working with non-infected patients was conducted using mean values of variables with a normal distribution with the application of the Student’s t-test for independent groups, average values of variables with a distribution different from the normal one using the Mann–Whitney test for dependent variables measured at the interval level of measurement, and the Kolmogorov–Smirnov test for dependent variables measured at the ordinal level of measurement. When the difference between groups was significant, the exact value of test probability was given, and if there were no significant differences, the abbreviation “p-NS” was used. The strength of the correlations between variables measured on at least ordinal levels of measurement was assessed by means of Spearman’s rho correlation coefficient (rho).

In all the analyses performed in the study, the existence of differences and the strength of correlations between variables were assessed at the significance level of *p* < 0.05.

## 3. Results

Among 151 surveyed nurses (100% women), more than half (52.3%) worked with SARS-CoV-2 infected patients, and the remainder (47.7%) worked with non-infected patients. The average age of nurses working with patients infected with SARS-CoV-2 virus was significantly lower than the average age of nurses working with non-infected patients (32.03 ± 8.94 vs. 41.04 ± 11.6, *p* < 0.001). The assessment of the financial situation differed considerably between the examined groups of nurses; the “very good” description was more common among nurses working with patients infected with SARS-CoV-2 than those who were working with patients not infected with SARS-CoV-2 (12.7% vs. 2.8%, *p* = 0.03). The distribution of work experience in the profession and the working time per month also differed significantly between the studied groups (respectively: *p* < 0.001 and *p* = 0.01). The nurses who worked with patients infected with the SARS-CoV-2 virus were twice as likely to have only a short work experience (≤5 years) in comparison to those working with the uninfected patients (44.3% vs. 22.2%), while a greater percentage of nurses working with patients infected with the SARS-CoV-2 virus worked from 160 to 200 h a month when compared to those who worked with uninfected patients (68.4% vs. 45.8%, *p* = 0.01)—Table 1.

The level of stress perceived by nurses working with infected patients assessed using the Perceived Stress Scale (PSS-10) was significantly higher than among nurses working with patients without SARS-CoV-2 infection (global score: 22.22 ± 5.94 vs. 20.21 ± 5.68, *p* = 0.03).

The most numerous group among nurses working with both infected and non-infected patients was made up of nurses who perceived a high intensity of stress (7–10 sten scores: 65.8% vs. 51.4%). The nurses who worked with non-infected patients were usually more likely to perceive average (5–6 sten scores: 36.15% vs. 26.6%) or low (1–4 sten scores: 12.5% vs. 7.6%, *p* = 0.50) intensity of stress than the nurses working with infected patients (the result according to sten scale: 7.22 ± 1.71 vs. 6.63 ± 1.72, *p* = 0.03) (Table 2).

No significant correlations were found between the intensity of perceived stress and nurses’ age, both in the case of nurses working with SARS-CoV-2-infected patients and those working with non-infected ones.

According to the Mini-COPE coping inventory, nurses working with patients infected with the SARS-CoV-2 virus usually chose strategies focused on the problem, such as active coping (taking actions aimed at improving the situation) or seeking instrumental support (looking for and receiving advice and help from others), as well as strategies focused on emotions (i.e., seeking emotional support (looking for encouragement, understanding or support from others)), whereas nurses working with non-infected patients most frequently chose coping strategies focused on the problem (i.e., active coping and planning (wondering and planning what should be done)).

The least frequent strategies chosen by both nurses working with patients infected with SARS-CoV-2 and nurses working with non-infected ones included substance use (taking psychoactive substances in order to alleviate unpleasant emotions), sense of humor (joking and treating the situation like fun) and behavioral disengagement (resigning from efforts to achieve the goal). A statistically significant difference was observed for the strategy of self-distraction (engaging in other activities so as not to think about unpleasant situations), which was on average more frequent among nurses working with patients infected with the SARS-CoV-2 virus than among nurses working with non-infected patients (1.93 vs. 1.66, *p* = 0.01) (Table 3).

An analysis of Spearman’s rho correlation coefficient in the examined group showed a significantly negative correlation between respondents’ age and venting emotions (they did not reveal negative emotions) and self-blame (they did not criticize and blame themselves for what had happened) in the group of nurses working with patients infected with the SARS-CoV-2 virus, and denial (denying that something had happened) and substance use (they did not take psychoactive substances in order to alleviate negative emotions) in the group of nurses working with patients not infected with the SARS-CoV-2 virus. A significantly positive correlation was also observed between nurses’ age and denial (they did not deny that something had happened) in the group of nurses working with patients infected with the SARS-CoV-2 virus (Table 4).

An analysis of Spearman’s rho correlations coefficient showed a significantly negative correlation between the intensity of perceived stress and positive reframing (they perceived the situation in a more negative light) among nurses working with patients infected with the SARS-CoV-2 virus and seeking emotional support (they did not look for encouragement, understanding or support from others) among nurses working with patients not infected with the SARS-CoV-2 virus. A significantly positive correlation was found between self-blame (criticizing and blaming themselves for what had happened) and the intensity of perceived stress among nurses working with both SARS-CoV-2-infected and non-infected patients (Table 5).

## 4. Discussion

Working during the COVID-19 pandemic is a particular challenge for nurses because, while performing their daily routines, they put their life and health at risk, which is accompanied by intensified stress [1,2]. There are few reports in the scientific literature on the assessment of the intensity of stress and coping strategies used by Polish nurses working with patients infected and not infected with the SARS-CoV-2 virus during the COVID-19 pandemic. Most of the studies that have been conducted so far have a selective character and do not take into account particular professional groups within healthcare staff or direct contact with patients infected and not infected with the SARS-CoV-2 virus during the COVID-19 pandemic.

This study, conducted between January and March 2021, shows that nurses working with patients infected with the virus were younger and at the same time had shorter professional experience than nurses working with patients not infected with the SARS-CoV-2 virus. As the wards where SARS-CoV-2 patients were treated were heavily understaffed, nurses were forced to do overtime. The studies conducted by Sagherian et al. in the United States also confirmed that nurses worked above standard working hours during the COVID-19 pandemic [16].

The authors’ own study shows that the level of perceived stress assessed by means of the PSS scale was higher among nurses working with infected patients than among nurses working with patients without SARS-CoV-2 infection (22.22 vs. 20.21). Comparable results were obtained by Sagherian et al. in the United States in their studies examining nurses working with patients infected with the SARS-CoV-2 virus and those working with non-infected patients; the nurses who worked with infected patients were more likely to perceive a higher level of stress and suffer from insomnia, chronic fatigue, post-traumatic stress disorder or mental stress than nurses working with non-infected patients [16]. Differences in the level of stress between healthcare workers taking care of patients infected with the SARS-CoV-2 virus and those working with non-infected patients were also observed in the study conducted in Italy by Trumello et al. Their study showed that the level of stress assessed, just like in the authors’ own study, by means of PSS-10 reached 19.78 in the group of healthcare staff working with patients infected with the virus, whereas in the group taking care of patients not infected with SARS-CoV-2, the stress level was 17.82 [17], which indicated an average intensity of stress in both groups. Moreover, the study showed that the staff working with patients infected with the SARS-CoV-2 virus reported a higher level of occupational burnout and lower level of job satisfaction than the staff working with patients not infected with the SARS-CoV-2 virus [17]. Other studies also confirmed a high intensity of stress (24 according to PSS-10), as well as symptoms of depression, anxiety and insomnia among healthcare staff working with patients infected with SARS-CoV-2 [18,19,20], especially among women [21,22,23], which tended to increase along with respondents’ age [24]. No relationship between age and the level of intensity of stress was found in the authors’ own study.

The COVID-19 pandemic not only had an impact on the intensity of stress among nurses working with patients infected and not infected with the SARS-CoV-2 virus but also changed and diversified their strategies of coping with stress. This study shows that in the group of nurses working with patients infected with the SARS-CoV-2 virus, the most frequently chosen strategies of coping with stress assessed according to the Mini-COPE stress inventory were strategies focused on the problem, such as active coping (i.e., taking actions aimed at improving the situation) and seeking instrumental support (i.e., looking for and receiving advice and help from others), as well as emotion-focused strategies such as seeking emotional support (i.e., looking for encouragement, understanding and support from others), whereas nurses working with non-infected patients had a tendency to choose only problem focused strategies, such as active coping or planning (i.e., wondering and planning what should be done).

The results of the systematic review and meta-analysis of the study prove, just like the study itself, that healthcare workers during the peak of the COVID-19 pandemic coped with stress using both coping strategies focused on the problem and strategies focused on emotions. Moreover, in this review, valuable evidence was found that showed the value and effectiveness of coping mechanisms, mental resilience and social support in maintaining mental health and psychological well-being among healthcare staff during the COVID-19 pandemic [25]. The results of the systematic review and meta-analysis do not take into account the nurses’ work before the COVID-19 pandemic with non-infected patients.

The least frequent strategies chosen by both nurses working with patients infected with SARS-CoV-2 and nurses working with non-infected ones included substance use (taking psychoactive substances in order to alleviate unpleasant emotions), sense of humor (joking and treating the situation like fun) and behavioral disengagement (resigning from efforts to achieve the goal). A statistically significant difference was found for the strategy of self-distraction, i.e., engaging in other activities so as not to think about an unpleasant situation, which was on average more frequent among nurses working with patients infected with SARS-CoV-2 virus than among nurses working with non-infected patients.

Studies conducted among Polish nurses in the period before the COVID-19 pandemic showed that the most frequent strategies of coping with various stressors included active coping, planning, self-distraction, seeking emotional support, positive reframing and progress. On the other hand, the least frequent strategies included denial, sense of humor, behavioral disengagement, substance use and seeking specialist help [26,27]. Although the aforementioned studies do not present the strategies of coping with stress during the COVID-19 pandemic that are applied by nurses working with patients infected with SARS-CoV-2 and with patients not infected with the virus, they are of considerable importance because they indicate the strategies that have been used before and might turn out to be insufficient. The studies conducted among medical staff in Hong Kong in 2004 and 2005 during the SARS epidemic show that the most frequently chosen strategies were the ones focused on the problem rather than those focused on emotions [28].

A strength of this study is that it was conducted in a group of nurses, who, unquestionably, constitute the biggest professional group among healthcare workers (HCW). The presented study is one of the few studies conducted in Poland that aimed at assessing occupational stress and coping strategies used by nurses working with patients infected and not infected with SARS-CoV-2 virus during the COVID-19 pandemic. Nevertheless, our results confirm the importance of further studies on identifying the differences in stress and coping strategies used by nurses working with SARS-CoV-2 infected patients and nurses working with non-infected patients during the COVID-19 pandemic. The results of this study imply that nurses working during the COVID-19 pandemic should be provided with psychological help and that support groups should be made available in order to minimize the development of posttraumatic stress disorder (PTSD).

**Limitations of the study**

It should be emphasized that the presented study has some limitations. It is a cross-sectional study that was relatively short, as it was conducted between January and March 2021. However, mental stress might accumulate with time and have an impact on developing post-traumatic stress disorder (PTSD), which should be examined in further studies. A weakness of the study is the relatively small size of the examined group, which makes it impossible to analyze the phenomenon in detail, taking into account not only gender but also the position held or work experience. Therefore, subsequent studies ought to include a larger group of nurses, as well as representatives of other medical professions. Moreover, because of its nature (snowball sampling), the study was conducted only in a group of people using information and communication technologies.

## 5. Conclusions

During the COVID-19 pandemic, nurses working with SARS-CoV-2 patients experience more intense stress than those working with non-infected patients. Nurses working with SARS-CoV-2 patients tended to cope with stress using strategies focused on the problem (active coping, seeking instrumental support) and on emotions (seeking encouragement, understanding and support of other people), while those working with non-infected patients were likely to choose strategies focused only on the problem (active coping and planning). Moreover, the study showed a relationship between age and strategies of coping with stress.

## Figures and Tables

**Table 1:** Socio-demographic characteristics of nurses working with patients infected and those working with patients not infected with the SARS-CoV-2 virus.

Socio-Demographic Variables		Nurses Working with Patients Infected with SARS-CoV-2 Virus:		*p*
		Yes N = 79 (52.3%)	No N = 72 (47.7%)	
Age (in years)	Mean ± SD	32.03 ± 8.94	41.04 ± 11.6	˂0.001 ***
	Min.–Max.	22–53	22–61	
Education N (%)	medical highschool/vocational college/Bachelor’s degree in nursing	32 (40.5)	32 (44.4)	0.625
	Master’s degree in nursing	47 (59.5)	40 (55.6)	
Marital status N (%)	single/widow/divorced	25 (31.6)	19 (26.4)	0.478
	married/informal relationship	54 (68.4)	53 (73.6)	
Financial status N (%)	average	10 (12.7)	16 (22.2)	0.036 *
	good	59 (74.7)	54 (75.0)	
	very good	10 (12.7)	2 (2.8)	
Work experience in nursing profession (in years)	≤5	35 (44.3)	16 (22.2)	<0.001 ***
	6–10	20 (25.3)	9 (12.5)	
	11–20	11 (13.9)	11 (15.3)	
	>20	13 (16.5)	36 (50.0)	
Number of working hours (monthly)	˂160	14 (17.7)	27 (37.5)	0.012 *
	160–200	54 (68.4)	33 (45.8)	
	>200	11 (13.9)	12 (16.7)	

SD—standard deviation, Min.—minimum, Max.—maximum, N—number of respondents, %—percentage of respondents, * *p* < 0.05, *** *p* < 0.001.

**Table 2:** Intensity of perceived stress in nurses working with patients infected and those working with patients not infected with the SARS-CoV-2 virus.

Intensity of Perceived Stress (PSS-10)		Nurses Working with Patients Infected with SARS-CoV-2 Virus:		*p*
		Yes	No	
Global score	Mean ± SD	22.22 ± 5.94	20.21 ± 5.68	0.036 *
	Min.–Max.	9–38	6–34	
According to sten scale	Mean ± SD	7.22 ± 1.71	6.63 ± 1.72	0.036 *
	Min.–Max.	3–10	3–10	

SD—standard deviation, Min.—minimum, Max.—maximum, * *p* < 0.05.

**Table 3:** Strategies of coping with stress used by nurses working with patients infected and those working with patients not infected with the SARS-CoV-2 virus.

Strategies of Coping with Stress (Mini-COPE)	Nurses Working with Patients Infected with the SARS-CoV-2 Virus		*p*
	Yes	No	
	Mean ± SD		
Active coping	2.07 ± 0.54	2.11 ± 0.58	0.702
Planning	1.97 ± 0.57	2.02 ± 0.59	0.630
Positive reframing	1.67 ± 0.68	1.68 ± 0.64	0.976
Acceptance	1.80 ± 0.57	1.81 ± 0.63	0.874
Sense of humor	0.86 ± 0.55	0.79 ± 0.60	0.513
Seeking emotional support	2.01 ± 0.69	1.88 ± 0.70	0.254
Seeking instrumental support	2.00 ± 0.62	1.87 ± 0.65	0.210
Self-distraction	1.93 ± 0.64	1.66 ± 0.66	0.012 *
Denial	1.11 ± 0.58	1.03 ± 0.68	0.445
Venting	1.34 ± 0.51	1.27 ± 0.53	0.365
Self-blame	1.21 ± 0.73	1.15 ± 0.82	0.623
	Median (Q1–Q3)		*p*
Turning to religion	1.0 (0.5–2.0)	1.5 (1.0–2.0)	0.825
Substance use	0.0 (0.0–1.0)	0.0 (0.0–1.0)	0.249
Behavioral disengagement	1.0 (0.0–1.0)	0.5 (0.0–1.0)	0.545

SD—standard deviation, Q1—lower quartile, Q3—upper quartile, * *p* < 0.05.

**Table 4:** Spearman’s rank correlation coefficient between strategies of coping with stress and nurses’ age.

Strategies of Coping with Stress (Mini-COPE)	Age (in Years)			
	Nurses Working with Patients Infected with the SARS-CoV-2 Virus			
	Yes		No	
	rho	*p*	rho	*p*
Active coping	0.16	0.142	0.03	0.752
Planning	0.03	0.767	0.09	0.413
Positive reframing	0.13	0.242	0.14	0.216
Acceptance	0.13	0.248	0.17	0.153
Sense of humor	0.04	0.695	−0.22	0.063
Turning to religion	0.08	0.456	0.20	0.083
Seeking emotional support	−0.08	0.438	−0.13	0.250
Seeking instrumental support	−0.13	0.231	−0.15	0.199
Self-distraction	−0.05	0.658	0.01	0.946
Denial	0.22	0.043 *	−0.22	0.054
Venting	−0.32	0.003 **	−0.06	0.574
Substance use	−0.15	0.167	−0.29	0.011 *
Behavioral disengagement	0.17	0.121	−0.19	0.110
Self-blame	−0.24	0.031 *	−0.15	0.188

* *p* < 0.05; ** *p* < 0.01.

**Table 5:** Strategies of coping with stress and the intensity of stress perceived by nurses working with patients infected and not infected with the SARS-CoV-2 virus.

Strategies of Coping with Stress (Mini-COPE)	Intensity of Perceived Stress (PSS-10)			
	Nurses Working with Patients Infected with the SARS-CoV-2 Virus			
	Yes		No	
	rho	*p*	rho	*p*
Active coping	−0.15	0.179	−0.21	0.067
Planning	−0.19	0.086	−0.10	0.382
Positive reframing	−0.27	0.015 *	−0.15	0.193
Acceptance	−0.13	0.234	−0.21	0.076
Sense of humor	0.02	0.821	0.07	0.540
Turning to religion	−0.05	0.613	0.20	0.078
Seeking emotional support	−0.07	0.525	−0.26	0.026 *
Seeking instrumental support	−0.03	0.748	−0.21	0.075
Self-distraction	−0.04	0.667	0.008	0.947
Denial	0.001	0.995	0.16	0.179
Venting	0.08	0.439	0.16	0.156
Substance use	0.05	0.627	0.13	0.250
Behavioral disengagement	0.16	0.137	0.22	0.054
Self-blame	0.31	0.005 **	0.42	0.000 ***

* *p* < 0.05, ** *p* < 0.01, *** *p* < 0.001.

## Data Availability

The datasets generated and analyzed during the current study are available from the corresponding authors on reasonable request.

## References

[1] Cai H., Tu B., Ma J., Chen L., Fu L., Jiang Y., Zhuang Q. (2020). Psychological Impact and Coping Strategies of Frontline Medical Staff in Hunan Between January and March 2020 During the Outbreak of Coronavirus Disease 2019 (COVID 19) in Hubei, China. Med. Sci. Monit..

[2] Tsamakis K., Rizos E., Manolis A.J., Chaidou S., Kympouropoulos S., Spartalis E., Spandidos D.A., Tsiptsios D., Triantafyllis A.S. (2020). COVID-19 pandemic and its impact on mental health of healthcare professionals. Exp. Med..

[3] Mo Y., Deng L., Zhang L., Lang Q., Liao C., Wang N., Qin M., Huang H. (2020). Work stress among Chinese nurses to support Wuhan in fighting against COVID-19 epidemic. J. Nurs. Manag..

[4] Kisely S., Warren N., McMahon L., Dalais C., Henry I., Siskind D. (2020). Occurrence, prevention, and management of the psychological effects of emerging virus outbreaks on healthcare workers: Rapid review and meta-analysis. BMJ.

[5] Nowicki G.J., Ślusarska B., Tucholska K., Naylor K., Chrzan-Rodak A., Niedorys B. (2020). The Severity of Traumatic Stress Associated with COVID-19 Pandemic, Perception of Support, Sense of Security, and Sense of Meaning in Life among Nurses: Research Protocol and Preliminary Results from Poland. Int. J. Env. Res. Public Health.

[6] Chandler-Jeanville S., Nohra R.G., Loizeau V., Lartigue-Malgouyres C., Zintchem R., Naudin D., Rothan-Tondeur M. (2021). Perceptions and Experiences of the COVID-19 Pandemic amongst Frontline Nurses and Their Relatives in France in Six Paradoxes: A Qualitative Study. Int. J. Env. Res. Public Health.

[7] Sygit-Kowalkowska E. (2014). Coping with stress as a health behavior—Psychological perspectives. Hygeia Public Health.

[8] Kupcewicz E., Jóźwik M. (2019). Positive Orientation and Strategies for Coping with Stress as Predictors of Professional Burnout among Polish Nurses. Int. J. Env. Res. Public Health.

[9] Huang L., Lei W., Xu F., Liu H., Yu L. (2020). Emotional responses and coping strategies in nurses and nursing students during COVID-19 outbreak: A comparative study. PLoS ONE.

[10] Juczyński Z., Ogińska-Bulik N. (2012). Narzędzia Pomiaru Stresu i Radzenia Sobie ze Stresem.

[11] Serwis Rzeczypospolitej Polskiej Raport Zakażeń Koronawirusem (SARS-CoV-2). https://www.gov.pl/web/koronawirus/wykaz-zarazen-koronawirusem-sars-cov-2.

[12] Ebert J.F., Huibers L., Christensen B., Christensen M.B. (2018). Paper- or Web-Based Questionnaire Invitations as a Method for Data Collection: Cross-Sectional Comparative Study of Differences in Response Rate, Completeness of Data, and Financial Cost. J. Med. Internet. Res..

[13] Uhlig C.E., Seitz B., Eter N., Promesberger J., Busse H. (2014). Efficiencies of Internet-based digital and paper-based scientific surveys and the estimated costs and time for different-sized cohorts. PLoS ONE.

[14] Arafa A.E., Anzengruber F., Mostafa A.M., Navarini A.A. (2019). Perspectives of online surveys in dermatology. J. Eur. Acad. Derm. Venereol..

[15] World Medical Association (2013). World Medical Association Declaration of Helsinki: Ethical principles for medical research involving human subjects. JAMA.

[16] Sagherian K., Steege L.M., Cobb S.J., Cho H. (2020). Insomnia, fatigue and psychosocial well-being during COVID-19 pandemic: A cross-sectional survey of hospital nursing staff in the United States. J. Clin. Nur..

[17] Trumello C., Bramanti S.M., Ballarotto G., Candelori C., Cerniglia L., Cimino S., Crudele M., Lombardi L., Pignataro S., Viceconti M.L. (2020). Psychological adjustment of healthcare workers in Italy during the COVID-19 pandemic: Differences in stress, anxiety, depression, burnout, secondary trauma, and compassion satisfaction between Frontline and Non-Frontline Professionals. International journal of environmental research and public health. Int. J. Env. Res. Public Health.

[18] Rossi R., Socci V., Pacitti F., Di Lorenzo G., Di Marco A., Siracusano A., Rossi A. (2020). Mental Health Outcomes Among Frontline and Second-Line Health Care Workers During the Coronavirus Disease 2019 (COVID-19) Pandemic in Italy. JAMA Netw. Open.

[19] Costa C., Teodoro M., Briguglio G., Vitale E., Giambò F., Indelicato G., Micali E., Italia S., Fenga C. (2021). Sleep Quality and Mood State in Resident Physicians during COVID-19 Pandemic. Int. J. Env. Res. Public Health.

[20] Zhan Y., Ma S., Jian X., Cao Y., Zhan X. (2020). The Current Situation and Influencing Factors of Job Stress Among Frontline Nurses Assisting in Wuhan in Fighting COVID-19. Front. Public Health.

[21] Lai J., Ma S., Wang Y., Cai Z., Hu J., Wei N., Wu J., Du H., Chen T., Li R. (2020). Factors Associated with Mental Health Outcomes Among Health Care Workers Exposed to Coronavirus Disease 2019. JAMA Netw. Open.

[22] Sánchez-Sánchez E., García-Álvarez J.Á., García-Marín E., Gutierrez-Serrano M., Alférez M.J.M., Ramirez-Vargas G. (2021). Impact of the COVID-19 Pandemic on the Mental Health of Nurses and Auxiliary Nursing Care Technicians—A Voluntary Online Survey. Int. J. Env. Res. Public Health.

[23] Hammond N.E., Crowe L., Abbenbroek B., Elliott R., Tian D.H., Donaldson L.H., Fitzgerald E., Flower O., Grattan S., Harris R. (2021). Impact of the coronavirus disease 2019 pandemic on critical care healthcare workers’ depression, anxiety, and stress levels. Aust. Crit. Care.

[24] Otgonbaatar D., Ts L., Ariunaa D., Tundevrentsen A., Naranbaatar N., Munkhkhand J. (2020). Occupational Stress in Nurse sAA —The Study Provided during the Urged Pandemic COVID-19 Quarantine Period. Psychology.

[25] Galanis P., Vraka I., Fragkou D., Bilali A., Kaitelidou D. (2021). Nurses’ burnout and associated risk factors during the COVID-19 pandemic: A systematic review and meta-analysis. J. Adv. Nurs..

[26] Siemianowska T., Podsiadły D., Ślusarz R. (2018). Occupational stress reactions among nurses working in behaviural wards. Innov. Nurs. Health Sci..

[27] Grochowska A., Bodys-Cupak I., Korus M. (2017). Ways to cope with the difficulties of nurses working in pediatric wards. Pielęg. Pol..

[28] Wong T.W., Yau J.K., Chan C.L., Kwong R.S., Ho S.M., Lau C.C., Lau F.L., Lit C.H. (2005). The psychological impact of severe acute respiratory syndrome outbreak on healthcare workers in emergency departments and how they cope. Eur. J. Emerg. Med..

